# Supplementary material for: Structural insights into regulation of the PEAK3 pseudokinase scaffold by 14-3-3
Source: Nat Commun. 2023 Jun 19;14:3543. doi: 10.1038/s41467-023-38864-0 (PMC10279700; doi:10.1038/s41467-023-38864-0)
Supplement: Supplementary file 1 — Supplementary Information [file 41467_2023_38864_MOESM1_ESM.pdf]

Supplementary Information.

Title: Structural insights into regulation of the PEAK3 pseudokinase scaffold by 14-3-3

Authors: Hayarpi Torosyan[1,2,†], Michael D. Paul[1,†], Antoine Forget[3,4], Megan Lo[1], Devan Diwanji[1,5], Krzysztof Pawłowski[6,7], Nevan J. Krogan[3,4,8], Natalia Jura[1,3,4,*] & Kliment A. Verba[3,4,*]

[1] Cardiovascular Research Institute, University of California San Francisco, San Francisco, CA 94158, USA

[2]Biophysics Graduate Program, University of California San Francisco, San Francisco, CA 94158, USA

[3]Quantitative Biosciences Institute (QBI), University of California San Francisco, San Francisco, CA 94158, USA

[4]Department of Cellular and Molecular Pharmacology, University of California San Francisco, San Francisco, CA 94158, USA

[5]Medical Scientist Training Program, University of California San Francisco, San Francisco, CA 94158, USA

[6]Department of Molecular Biology, University of Texas Southwestern Medical Center, Dallas, TX 75390, USA

[7]Department of Biochemistry and Microbiology, Warsaw University of Life Sciences, 02-787 Warszawa, Poland

[8]J. David Gladstone Institutes, San Francisco, CA 94158

[†]Authors contributed equally to the work

[*]Correspondence should be addressed to K.A.V. (kliment.verba@ucsf.edu) or N.J. (natalia.jura@ucsf.edu)

Supplementary Figure 1.

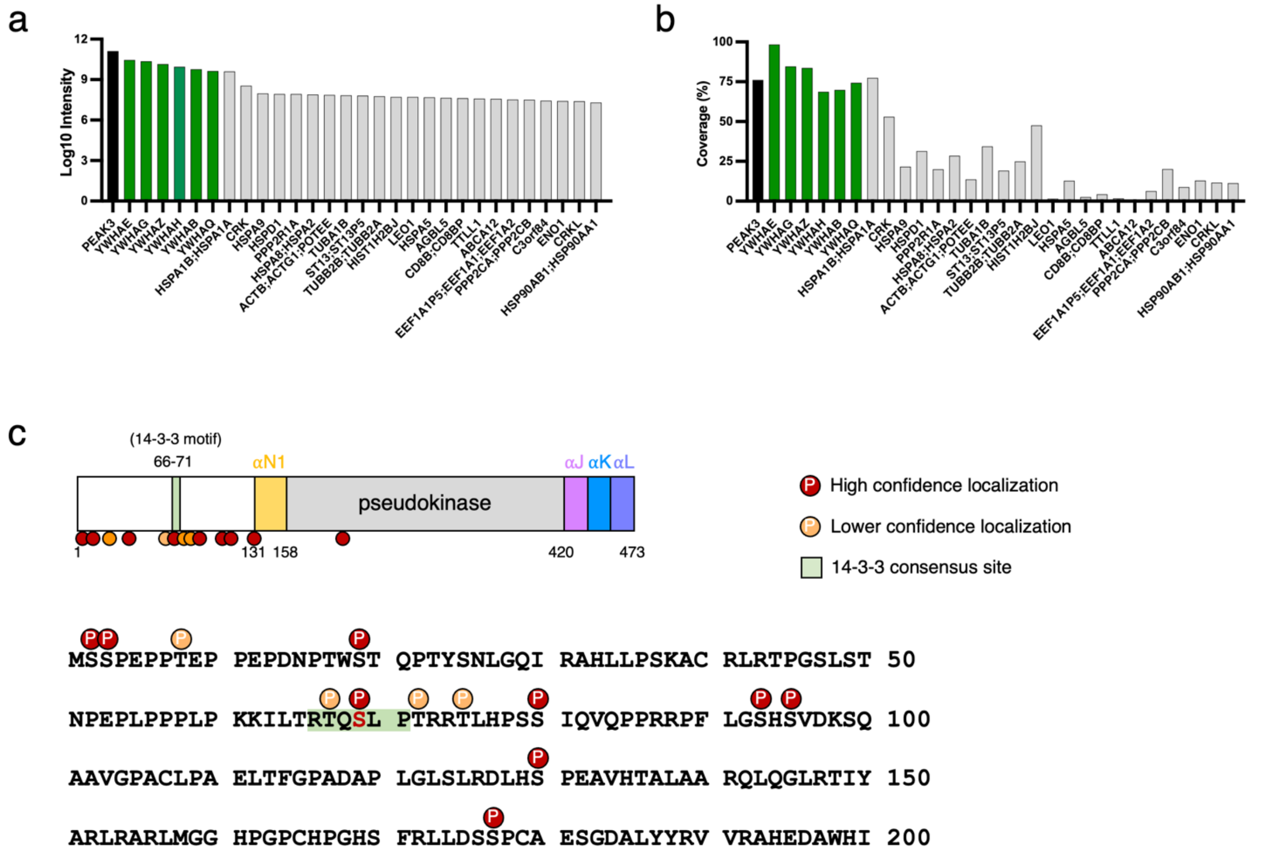

Supplementary Figure 1: Mass spectrometry analysis of the purified PEAK3/14-3-3 complex

a Bar chart representing the log10 protein spectral intensity for the top 30 proteins identified by mass spectrometry (MS) analysis of the purified PEAK3/14-3-3 complex, and **b** their corresponding protein sequence coverage (black: PEAK3, green: 14-3-3, gray: other interactors). **c** Schematic representation of the identified phospho-sites in PEAK3 by MS analysis (red: localization probability > 0.8; orange: localization probability < 0.8). The S69 phospho-site within the 14-3-3 consensus binding site (green) of PEAK3 represents a high confidence site.

Supplementary Figure 2.

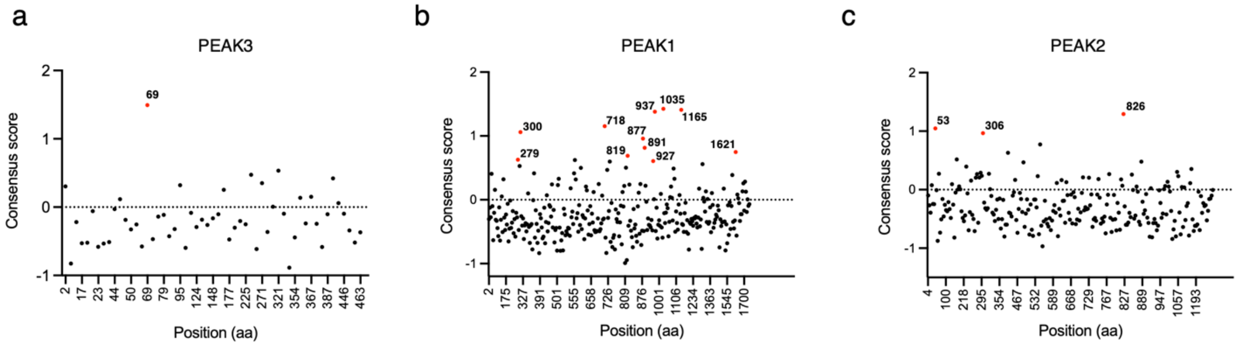

Position	Sequence
69	ILTRTQ[S]LPTR

Position	Sequence
279	ANFRAN[T]LSPV
300	IPLRNK[S]LQRI
718	CLNRGQ[S]SPQR
819	TPVRPK[S]LFTS
877	PPPRST[S]SPYH
891	LLQRHF[T]NWTK
927	KPKRWI[S]FKSF
937	FFRRRK[T]DEED
1035	EKKRSH[S]SPSQ
1165	MIIRAN[T]EPIS
1621	LKEREY[T]RADL

Position	Sequence
53	PQPRAG[S]LPPP
306	QEKRGP[S]FPKE
826	IVSRAA[S]SPDG

Supplementary Figure 2: Putative 14-3-3 binding sites in PEAK family members

a-c Sequence-based prediction of 14-3-3 consensus binding sites in PEAK family members using the 14-3-3-Pred webserver[1] based on three different classifiers (ANN, PSSM and SVM). Data points represent the amino acid position of the phosphorylated serine or threonine within the putative binding site. Sites, which score highly in all three prediction models, are colored red.

Supplementary Figure 3.

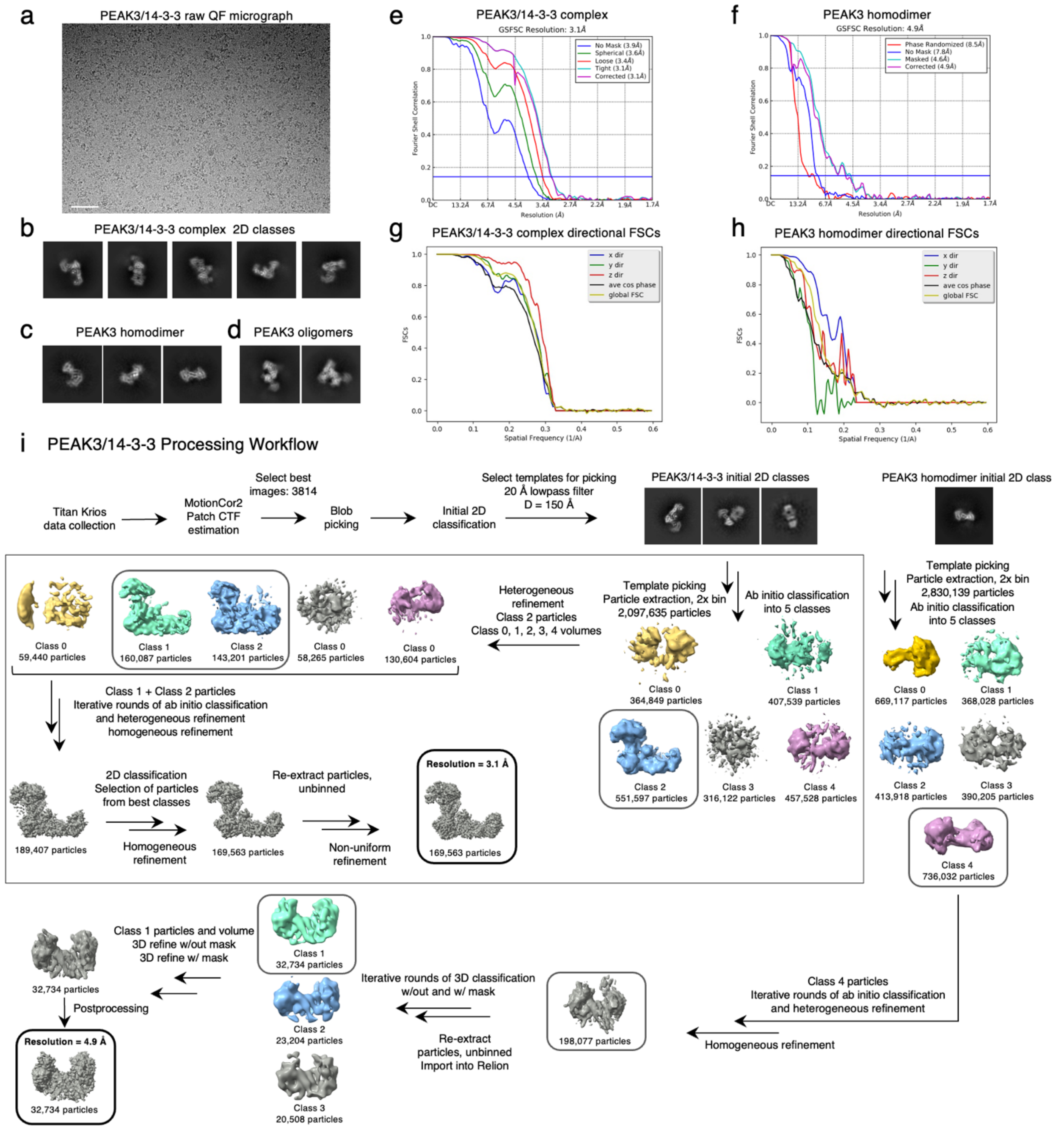

Supplementary Figure 3: Processing workflow, resolution estimation and map quality of PEAK3/14-3-3 complex and PEAK3 homodimer dataset

a Representative micrograph of the PEAK3/14-3-3 complex sample on Quantifoil R1.2/1.3 300 mesh Au holey-carbon grids, from a dataset with 3814 micrographs. The scale bar corresponds to

500 Å. **b-d** Example cryo-EM 2D class averages of particles corresponding to **(b)** PEAK3/14-3-3 complex, (**c)** PEAK3 homodimer, and **(d)** PEAK3 oligomers in the absence or presence of 14-3-3 binding. **e,f** Gold Standard Fourier Shell Correlation (GSFSC) of the final map used for model building of the (**e**) PEAK3/14-3-3 complex from CryoSPARC[2] with a reported resolution of 3.1 Å and final map for model building of the (**f**) PEAK3 homodimer from RELION[3] with a reported resolution of 4.9 Å. **g,h** Directional FSCs of the (**g**) PEAK3/14-3-3 complex and the (**h**) PEAK3 homodimer calculated by 3DFSC server[4]. **i** Workflow for processing the PEAK3/14-3-3 complex dataset. Gray boxes indicate model and associated particle stack used for downstream processing. The final model is indicated with a bolded black box.

Supplementary Figure 4.

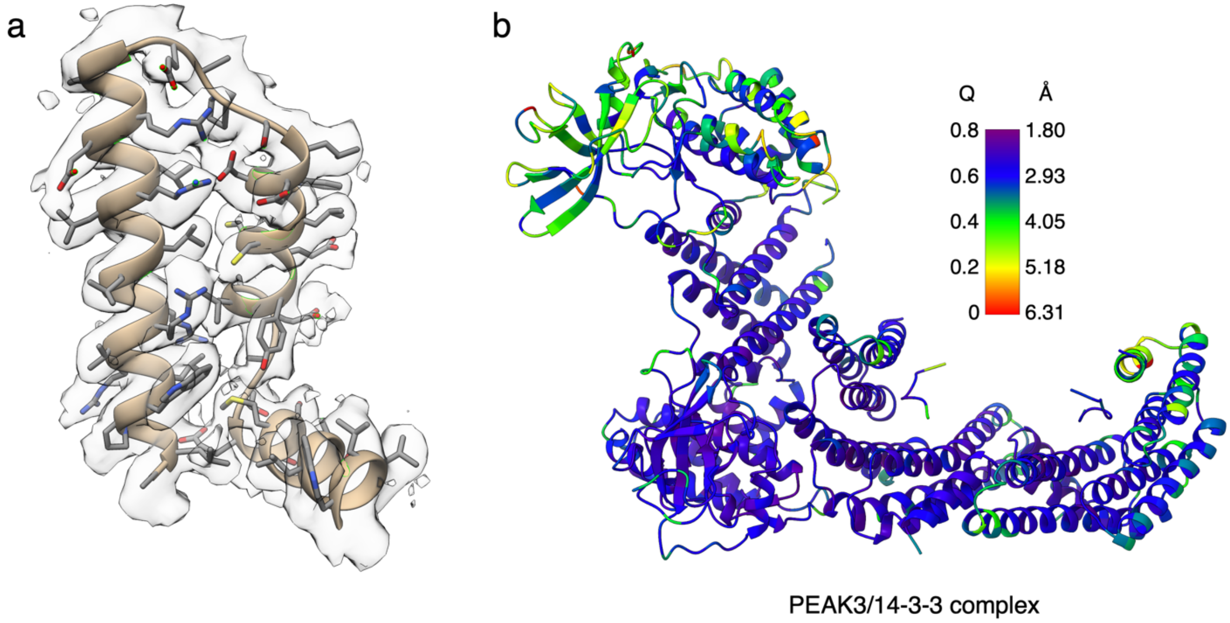

Supplementary Figure 4: Q-score analysis of the PEAK3/14-3-3 cryo-EM structure

a Zoomed-in view of the cryo-EM map and corresponding model of the PEAK3/14-3-3 complex (residues 419-472) demonstrating features appropriate for reported resolution. **b** PEAK3/14-3-3 complex model colored by estimated per residue Q-score. Color scale bar indicates corresponding estimated resolution in Å for reported Q-scores. Expected Q-score for a 3.1 Å structure is 0.569.

Supplementary Figure 5.

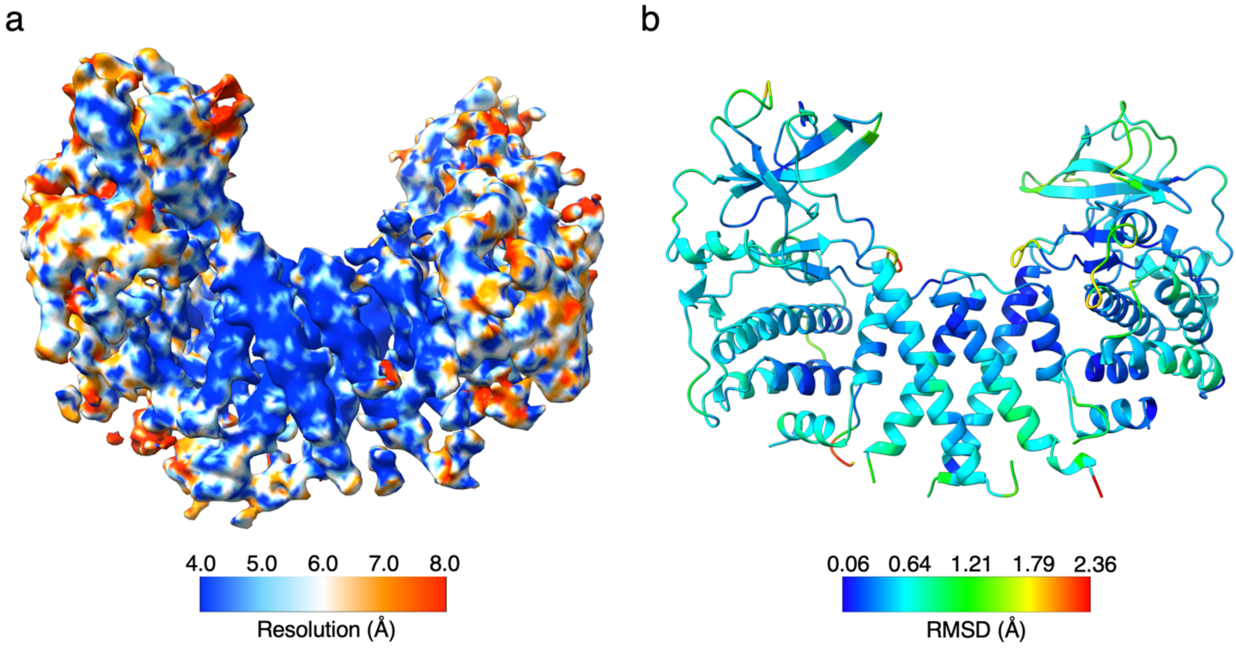

Supplementary Figure 5: Low-resolution structure of the PEAK3 homodimer

a Cryo-EM map of the PEAK3 homodimer colored according to local resolution determined by ResMap[5]. **b** Corresponding model of the PEAK3 homodimer structure colored by per residue Root Mean Square Deviation (RMSD, Å) relative to PEAK3 homodimer as part of the PEAK3/14-3-3 complex.

Supplementary Figure 6.

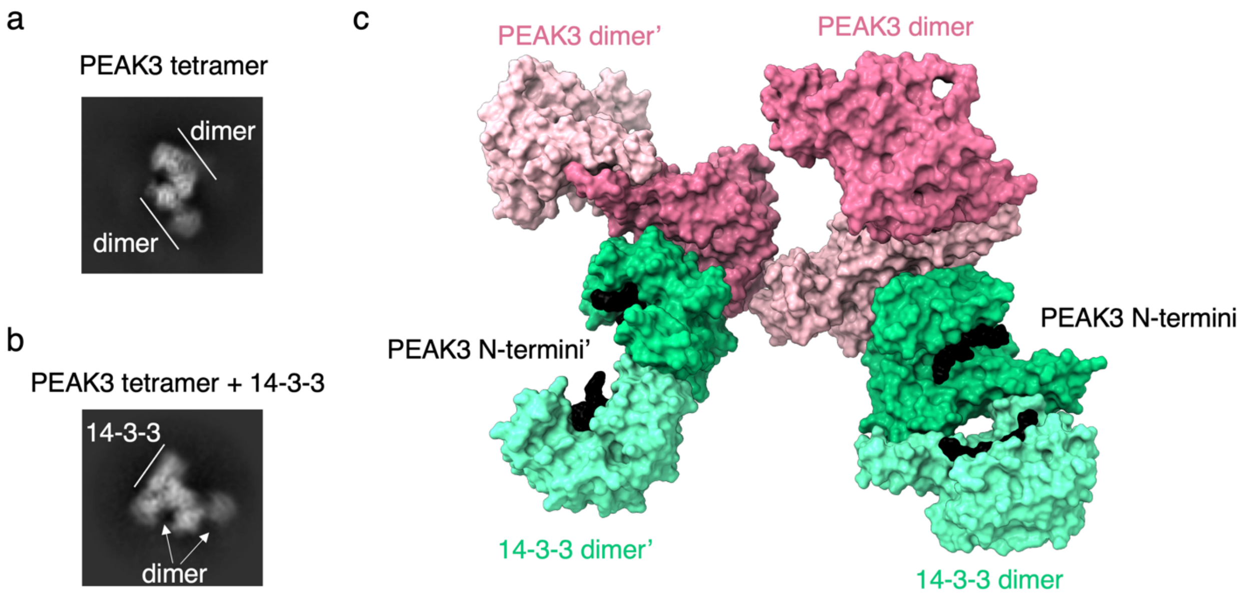

Supplementary Figure 6: PEAK3 oligomerization permits 14-3-3 binding

a,b PEAK3 oligomerization in the absence (**a**) and presence (**b**) of 14-3-3 binding as demonstrated by cryo-EM 2D class averages. **c** Putative oligomeric PEAK3/14-3-3 complex model derived from oligomeric interface identified from crystal packing of PEAK2 crystal structure (PDB ID: 5VE6). PEAK3 homodimer within the PEAK3/14-3-3 complex was first aligned with one of the two PEAK2 homodimers, followed by alignment of a second PEAK3 homodimer to the second PEAK2 homodimer.

Supplementary Figure 7.

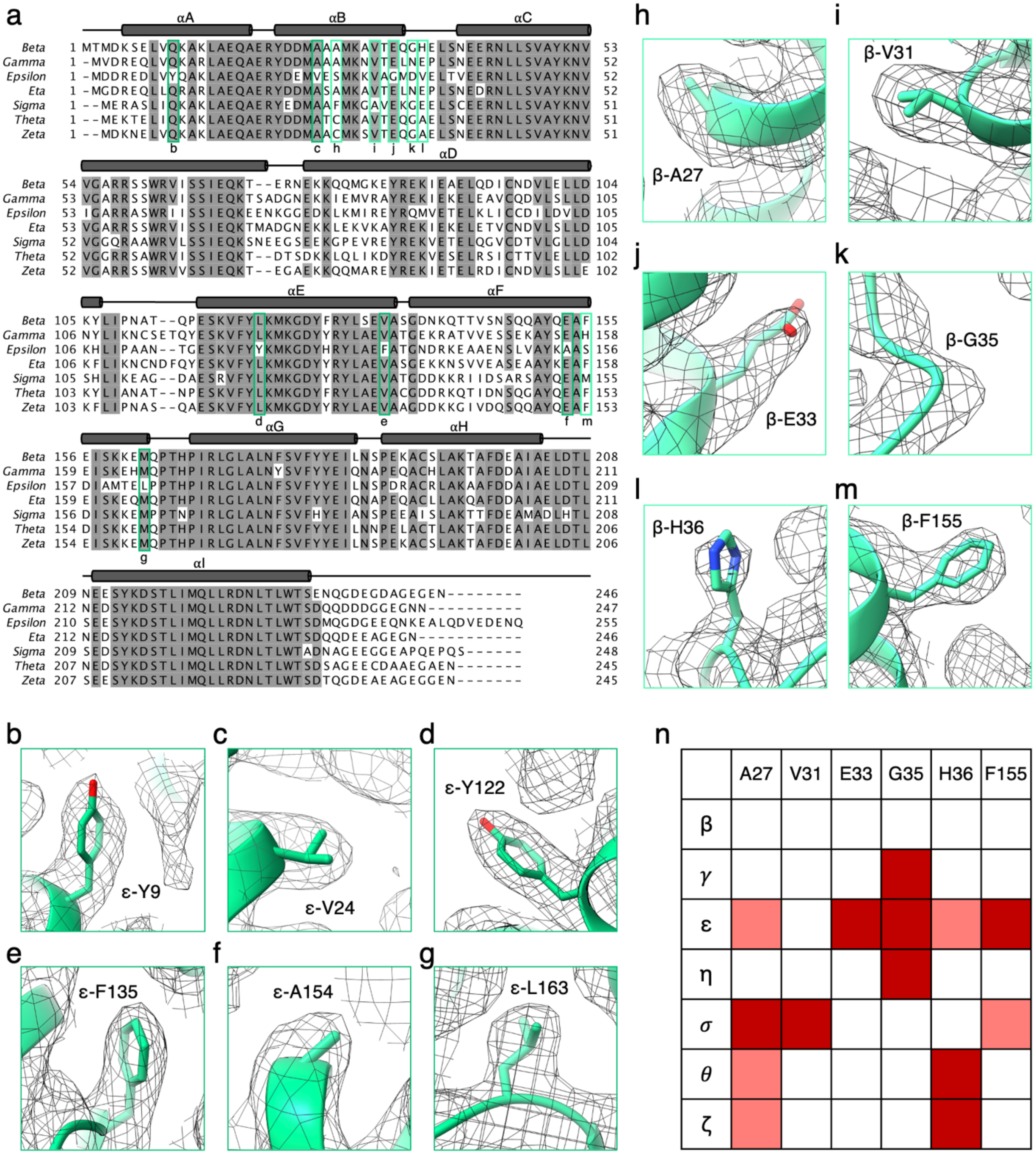

Supplementary Figure 7: Identification of 14-3-3 isoforms in the PEAK3/14-3-3 complex

a Structure-based sequence alignment of human 14-3-3 isoforms depicting their secondary structure elements. Conserved residues are shaded in gray. **b-m** Zoomed-in view of residue side chains in 14-3-3ε (**b-g**) and in 14-3-3β (**h-m**) overlayed with the cryo-EM map used to determine

the identity of the 14-3-3 monomers in the PEAK3/14-3-3 structure. **n** Summary table of (**h-m**) residues and their compatibility with the cryo-EM map in all 14-3-3 isoforms. Dark red, light red and white squares indicate that the corresponding residue in a particular 14-3-3 isoform is not consistent, somewhat consistent or consistent with the cryo-EM density, respectively.

Supplementary Figure 8.

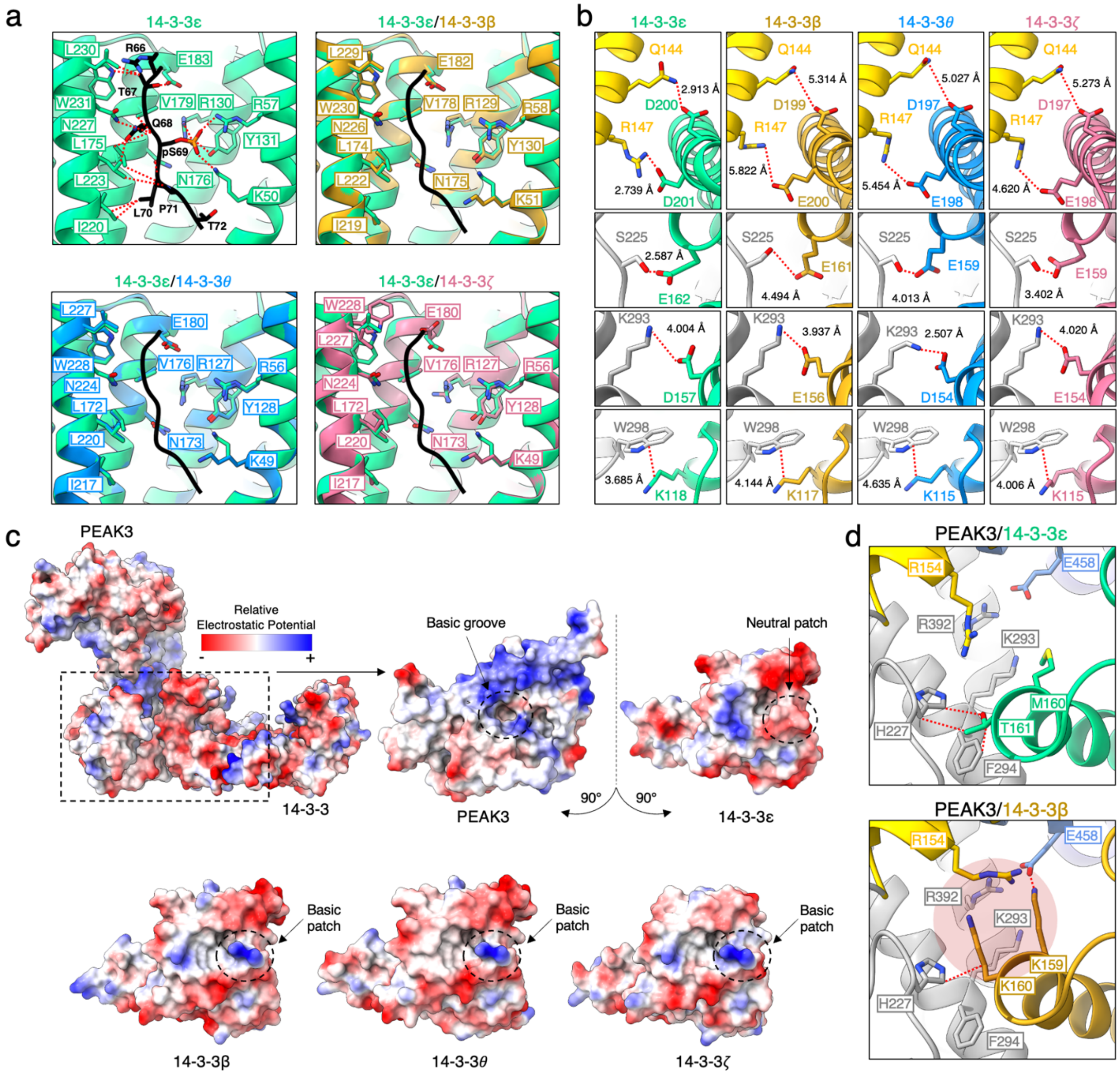

Supplementary Figure 8: Structural analysis of PEAK3's specificity for 14-3-3ε

The PEAK3 homodimer model from the PEAK3/14-3-3 complex and crystal structures of 14-3-3 β (PDB ID: 2C23), θ (PDB ID: 6KZH), and ζ (PDB ID: 1A38) were fit into the PEAK3/14-3-3 complex cryo-EM map with FastRelax protocol in Rosetta[6] in torsion space, and the resulting models were compared. **a** Top left: primary interface interactions between PEAK3 and 14-3-3ε. Top right and bottom left and right: corresponding overlays between 14-3-3ε (green) and 14-3-3β

(mustard yellow), 14-3-3θ (blue), and 14-3-3ζ (pink) demonstrating conservation of PEAK3-interacting residues among 14-3-3 isoforms. **b** Overlays of important secondary interface interactions found between PEAK3 and 14-3-3ε and preserved in 14-3-3β, θ, and ζ. **c** Surface representations of the PEAK3/14-3-3εβ complex and 14-3-3β, θ, and ζ colored by surface electrostatic potential, highlighting a distinct patch in 14-3-3ε which favors its interaction with PEAK3, relative to β, θ, and ζ isoforms. **d** Zoomed-in view of the patch identified in (**c**) highlighting key interactions between PEAK3 and 14-3-3ε (green) and PEAK3 and 14-3-3β (mustard yellow), with the red bubble emphasizing a charge clash between PEAK3 and 14-3-3β. Dashed lines in red indicate interactions with distances ≤ 4 Å, unless otherwise noted.

Supplementary Figure 9.

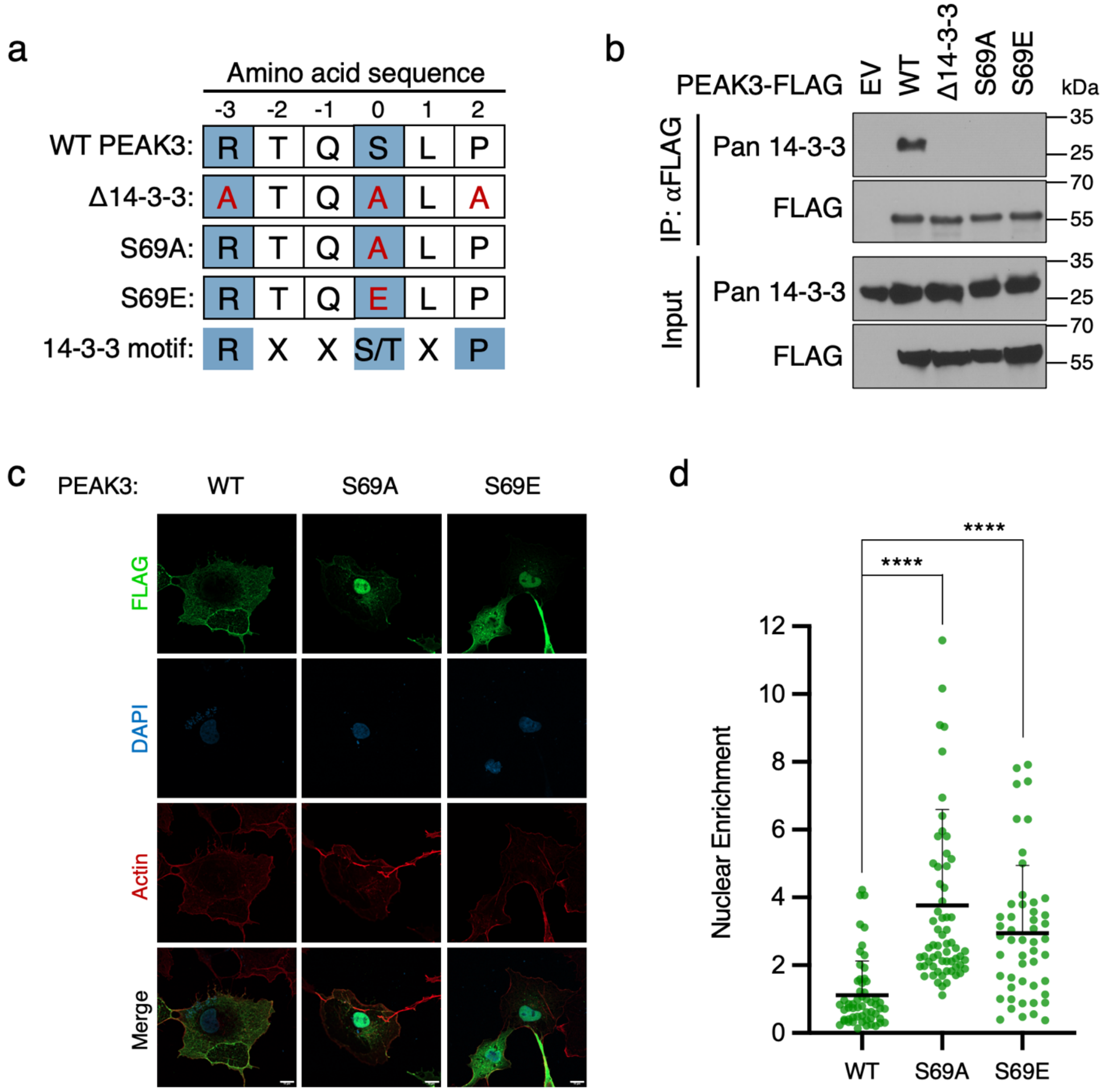

Supplementary Figure 9: A phosphomimetic substitution at S69 does not reproduce the PEAK3/14-3-3 interaction

a Diagram of PEAK3 mutants generated to probe PEAK3 binding to the primary site on 14-3-3. **b** Co-immunoprecipitation of endogenous 14-3-3 with FLAG-tagged WT PEAK3 and PEAK3 mutants (Δ14-3-3, S69A, S69E) transiently expressed in HEK293 cells. Co-immunoprecipitation data is representative of at least 3 independent experiments. **c** Immunofluorescence-based imaging

of FLAG-tagged PEAK3 transiently expressed in COS-7 cells. Representative confocal microscopy images show cells transfected with PEAK3 variants: WT, S69A or S69E. PEAK3 was detected with an anti-FLAG antibody (green), and cells were further stained with DAPI (blue, nucleus) and iFluor-647 conjugated phalloidin (red, actin). Scale bars: 10 µm. **d** Quantification of relative nuclear enrichment of PEAK3 under conditions of impaired 14-3-3 binding. The ratio of fluorescence intensity in the green channel after background subtraction measured in the nucleus to the fluorescence intensity of the non-nuclear portion of the cell is plotted for each PEAK3 variant; see Methods for details. Data are plotted as the mean with standard deviation, combining all cells from at least 3 independent experiments (n = 57, 65, and 50 total cells for WT, S69A, and S69E respectively). Statistical significance was determined using One-way ANOVA Dunnett's multiple comparisons test, ****$p<0.0001$. Source data are provided as a Source Data file.

Supplementary Figure 10.

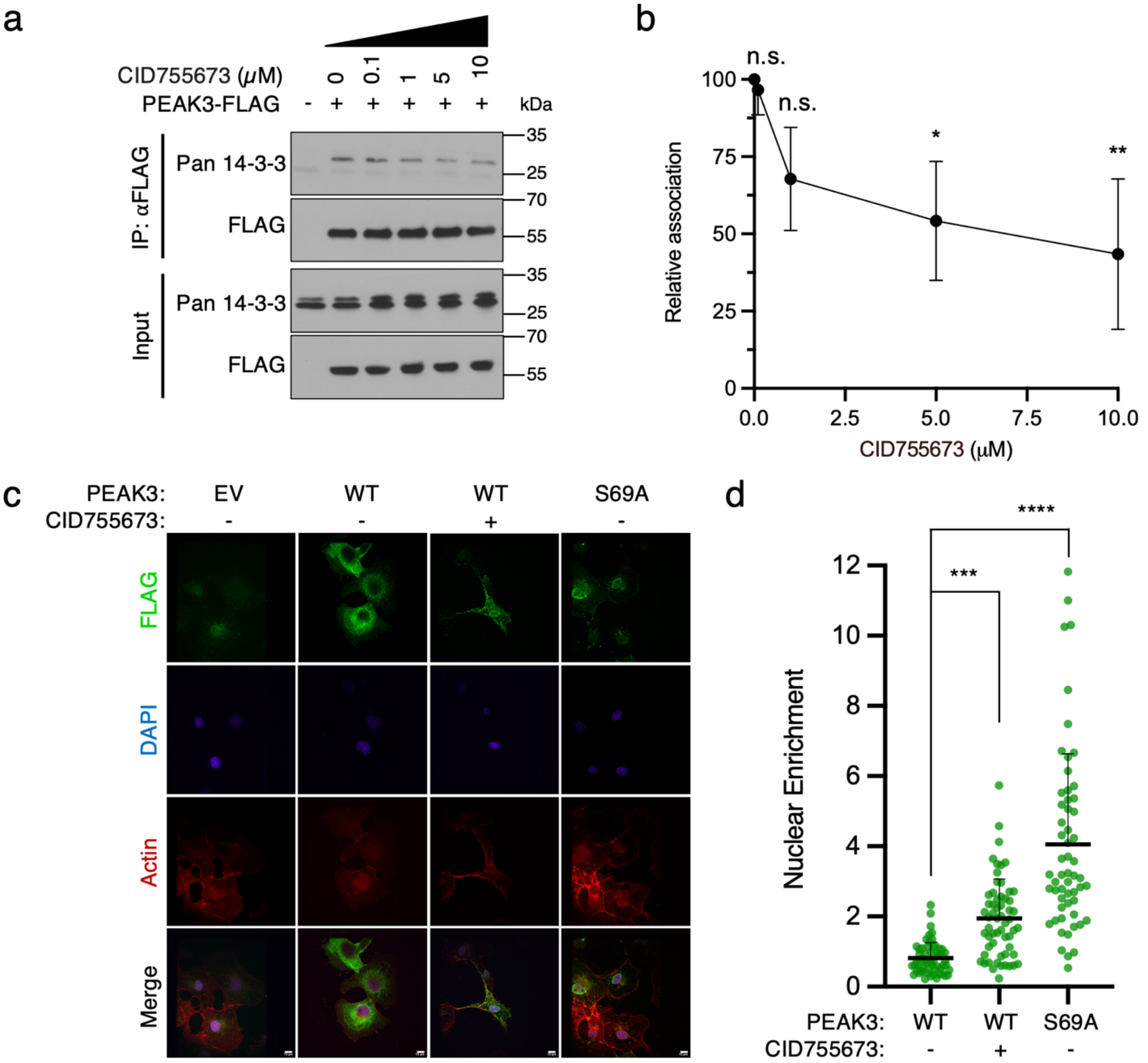

Supplementary Figure 10: Allosteric inhibition of PKD regulates the PEAK3/14-3-3 interaction

a Representative co-immunoprecipitation of endogenous 14-3-3 with FLAG-tagged WT PEAK3 transiently expressed in HEK293 cells treated with 0.1% DMSO or increasing concentrations of the allosteric PKD inhibitor CID755673. **b** Quantification of co-immunoprecipitation data shown in panel (**a**) plotted as the mean with standard deviation from 3 independent experiments.

Statistical significance was determined using One-way ANOVA Dunnett's multiple comparisons test, *$p < 0.05$, **$p < 0.01$. Protein levels were detected with the indicated antibodies. **c** Immunofluorescence-based imaging of FLAG-tagged PEAK3 transiently expressed in COS-7 cells. Representative confocal microscopy images show cells transfected with an empty vector (EV) control or indicated FLAG-tagged PEAK3 constructs treated with 0.1% DMSO or 10 μM PKD inhibitor CID755673. PEAK3 was detected with an anti-FLAG antibody (green), and cells were further stained with DAPI (blue, nucleus) and iFluor-647 conjugated phalloidin (red, actin). Scale bars: 10 μm. **d** Quantification of relative nuclear enrichment of PEAK3 under conditions of impaired 14-3-3 binding. The ratio of fluorescence intensity in the green channel after background subtraction measured in the nucleus to the fluorescence intensity of the non-nuclear portion of the cell is plotted for each PEAK3 variant; see Methods for details. Data are plotted as the mean with standard deviation, combining all cells from at least 3 independent experiments ($n = 58$, 59, and 57 total cells for WT, WT + CID755673, and S69A respectively). Statistical significance was determined using One-way ANOVA Dunnett's multiple comparisons test, ***$p<0.001$, ****$p<0.0001$. Source data are provided as a Source Data file.

Supplementary Table 1. Cryo-EM collection, refinement and resulting model statistics.

	PEAK3/14-3-3 complex (EMD-27630) (PDB 8DP5)	PEAK3 homodimer (EMD-27684) (PDB 8DS6)
**Data collection and processing**		
Magnification	105,000x	105,000x
Voltage (kV)	300	300
Total dose (e–/Å^2)	69	69
Dose rate (e-/physical pixel/sec)	16	16
Exposure per frame (sec)	0.025	0.025
Defocus range (μm)	-1.0 to -2.0	-1.0 to -2.0
Pixel size (Å)	0.835 (physical)	0.835 (physical)
Symmetry imposed	C1	C1
Initial particle images (no.)	2097635	2608418
Final particle images (no.)	169563	32734
Map resolution (Å)<br>FSC threshold (0.143)	3.1	4.9
Map resolution range (Å)	2.5-5.5	4.0-8.0
**Refinement**		
Initial model used (PDB code)	AF- Q6ZS72<br>AF- P62258<br>AF- P31946	AF- Q6ZS72

Model resolution (Å)<br>FSC threshold 0.5 (Masked)	3.3	6.8
Map sharpening *B* factor ($Å^2$)	-107.4	-105
Model composition<br>Non-hydrogen atoms<br>Protein residues	 8869<br>1145	 5030<br>669
B factors ($Å^2$)<br>Protein	 70.4	 265.5
R.M.S. deviations<br>Bond lengths (Å)<br>Bond angles (°)	 0.012<br>1.897	 0.012<br>1.514
Validation<br>MolProbity score<br>Clashscore<br>Poor rotamers (%)	 0.75<br>0.79<br>0.00	 0.61<br>0.30<br>0.00
Ramachandran plot<br>Favored (%)<br>Allowed (%)<br>Disallowed (%)	 98.93<br>1.07<br>0.00	 98.64<br>1.36<br>0.00

Supplementary References

1. Madeira, F. et al. 14-3-3-Pred: improved methods to predict 14-3-3-binding phosphopeptides. *Bioinformatics* **31**, 2276-2283 (2015).
2. Punjani, A., Rubinstein, J.L., Fleet, D.J. & Brubaker, M.A. cryoSPARC: algorithms for rapid unsupervised cryo-EM structure determination. *Nature methods* **14**, 290-296 (2017).
3. Zivanov, J. et al. New tools for automated high-resolution cryo-EM structure determination in RELION-3. *elife* **7**, e42166 (2018).
4. Tan, Y.Z. et al. Addressing preferred specimen orientation in single-particle cryo-EM through tilting. *Nature methods* **14**, 793-796 (2017).
5. Kucukelbir, A., Sigworth, F.J. & Tagare, H.D. Quantifying the local resolution of cryo-EM density maps. *Nature methods* **11**, 63-65 (2014).
6. DiMaio, F. et al. Atomic-accuracy models from 4.5-Å cryo-electron microscopy data with density-guided iterative local refinement. *Nature methods* **12**, 361-365 (2015).
