# Supplementary material for: Structural insights into regulation of the PEAK3 pseudokinase scaffold by 14-3-3
Source: Nat Commun. 2023 Jun 19;14:3543. doi: 10.1038/s41467-023-38864-0 (PMC10279700; doi:10.1038/s41467-023-38864-0)
Supplement: Supplementary file 2 — Reporting Summary [file 41467_2023_38864_MOESM2_ESM.pdf]

Corresponding author(s): Kliment A. Verba and Natalia Jura
Last updated by author(s): 05/09/2023

Reporting Summary

Nature Research wishes to improve the reproducibility of the work that we publish. This form provides structure for consistency and transparency in reporting. For further information on Nature Research policies, see our Editorial Policies and the Editorial Policy Checklist.

Statistics

For all statistical analyses, confirm that the following items are present in the figure legend, table legend, main text, or Methods section.

n/a	Confirmed	
☐	☒	The exact sample size (*n*) for each experimental group/condition, given as a discrete number and unit of measurement
☐	☒	A statement on whether measurements were taken from distinct samples or whether the same sample was measured repeatedly
☐	☒	The statistical test(s) used AND whether they are one- or two-sided<br>*Only common tests should be described solely by name; describe more complex techniques in the Methods section.*
☒	☐	A description of all covariates tested
☐	☒	A description of any assumptions or corrections, such as tests of normality and adjustment for multiple comparisons
☐	☒	A full description of the statistical parameters including central tendency (e.g. means) or other basic estimates (e.g. regression coefficient) AND variation (e.g. standard deviation) or associated estimates of uncertainty (e.g. confidence intervals)
☐	☒	For null hypothesis testing, the test statistic (e.g. F, t, r) with confidence intervals, effect sizes, degrees of freedom and P value noted<br>*Give P values as exact values whenever suitable.*
☒	☐	For Bayesian analysis, information on the choice of priors and Markov chain Monte Carlo settings
☒	☐	For hierarchical and complex designs, identification of the appropriate level for tests and full reporting of outcomes
☒	☐	Estimates of effect sizes (e.g. Cohen's d, Pearson's r), indicating how they were calculated

Our web collection on statistics for biologists contains articles on many of the points above.

Software and code

Policy information about availability of computer code

Data collection	SerialEM 4.0.3, Digital Micrograph 3.31.2359.0, Zen 2 blue edition v1.0 (Zeiss), Nikon Elements 5.02 build 1266, Q-Exactive Plus mass spectrometer
Data analysis	SCIPION 3.0.1, MotionCor2 v1.5.0, cryoSPARC v2.15.0, Relion 3.1, ChimeraX 1.2.5, UCSF Chimera 1.16, Rosetta 3.0, Coot 0.9.6, ISOLDE 1.2.1, Phenix 1.19.2, pyEM v0.5, ResMap v1.1.4, 3DFSC v3.0, Prism 9, FIJI v1.53f51 and v2.0.0, Uniprot, MaxQuant version 1.6.12.0, R v4.1.1 , SAINTexpress version 3.6.1, MMSEQ v1.0.11, Mafft v7, Weblogo 3, Cytoscape v3.8, STRING db v11.5

For manuscripts utilizing custom algorithms or software that are central to the research but not yet described in published literature, software must be made available to editors and reviewers. We strongly encourage code deposition in a community repository (e.g. GitHub). See the Nature Research guidelines for submitting code & software for further information.

Data

Policy information about availability of data

All manuscripts must include a data availability statement. This statement should provide the following information, where applicable:

- Accession codes, unique identifiers, or web links for publicly available datasets
- A list of figures that have associated raw data
- A description of any restrictions on data availability

The data that supports this study is available from the corresponding authors upon reasonable request. Cryo-EM maps have been deposited in the Electron Microscopy Data Bank (EMDB) under accession codes EMD-27630 (PEAK3/14-3-3) and EMD-27684 (PEAK3 homodimer). Associated models have been deposited in the Protein Data Bank (PDB) with accession codes 8DP5 and 8DS6, respectively. Models used for data analysis in this manuscript can be found in the PDB: 1ATP [https://doi.org/10.2210/pdb1ATP/pdb], 2O98 [https://doi.org/10.2210/pdb2O98/pdb], 5N6N [https://doi.org/10.2210/pdb5N6N/pdb], 6UAN [https://doi.org/10.2210/pdb6UAN/pdb], 6Q0K [https://doi.org/10.2210/pdb6Q0K/pdb], 7MFE [https://doi.org/10.2210/pdb7MFE/pdb], 7MFF [https://doi.org/10.2210/pdb7MFF/pdb], 1IB1 [https://doi.org/10.2210/pdb1IB1/pdb], 6XAG [https://doi.org/10.2210/pdb6XAG/pdb], 6U2H [https://doi.org/10.2210/pdb6U2H/pdb], 3AXY [https://doi.org/10.2210/pdb3AXY/pdb], 6GN0 [https://doi.org/10.2210/pdb6GN0/pdb], 6GNK [https://doi.org/10.2210/pdb6GNK/pdb], 6GNJ [https://doi.org/10.2210/pdb6GNJ/pdb], 6GN8 [https://doi.org/10.2210/pdb6GN8/pdb], 5LTW [https://doi.org/10.2210/pdb5LTW/pdb], 6GNN [https://doi.org/10.2210/pdb6GNN/pdb], 2C23 [https://doi.org/10.2210/pdb2C23/pdb], 6KZH [https://doi.org/10.2210/pdb6KZH/pdb], 1A38 [https://doi.org/10.2210/pdb1A38/pdb]. The mass spectrometry proteomics data have been deposited to the ProteomeXchange Consortium via the PRIDE partner repository with the dataset identifier PXD035574. Reviewers may access the dataset using the following credentials: Username of reviewer_pxd035574@ebi.ac.uk and password of VqfXfU8o. Source data for western blots (Figures 5, 7; Supplementary Figures 9, 10) , immunofluorescence (Figures 6, 7; Supplementary Figures 9, 10) and ADP-Glo kinase assay (Figure 7) are provided with this paper.

Field-specific reporting

Please select the one below that is the best fit for your research. If you are not sure, read the appropriate sections before making your selection.

☒ Life sciences ☐ Behavioural & social sciences ☐ Ecological, evolutionary & environmental sciences

For a reference copy of the document with all sections, see nature.com/documents/nr-reporting-summary-flat.pdf

Life sciences study design

All studies must disclose on these points even when the disclosure is negative.

Sample size	No sample size calculation was performed. The sample size was chosen based on the author's prior experiences with the experiments and previously published studies of similar design (PMIDs: 31311869, 35525557)
Data exclusions	No data were excluded to generate graphs.
Replication	Each experiment was repeated at least three times for reproducibility and all attempts were successful.
Randomization	This is not relevant for the types of assays reported in this study, because there was no allocation of experimental groups.
Blinding	These experiments did not reguire blinding. Each investigator performed a given experiment and labeled the corresponding samples and performed the analysis. All proper controls were included in the experimental design.

Reporting for specific materials, systems and methods

We require information from authors about some types of materials, experimental systems and methods used in many studies. Here, indicate whether each material, system or method listed is relevant to your study. If you are not sure if a list item applies to your research, read the appropriate section before selecting a response.

Materials & experimental systems

n/a	Involved in the study	
☐	☒	Antibodies
☐	☒	Eukaryotic cell lines
☒	☐	Palaeontology and archaeology
☒	☐	Animals and other organisms
☒	☐	Human research participants
☒	☐	Clinical data
☒	☐	Dual use research of concern

Methods

n/a	Involved in the study	
☒	☐	ChIP-seq
☒	☐	Flow cytometry
☒	☐	MRI-based neuroimaging

Antibodies

Antibodies used	The following primary antibodies were used: anti-FLAG (mouse, Sigma, F1804, 3 ug/10 cm plate), anti-HA (mouse, SCBT, sc-7392, 3 ug/10 cm plate), anti-FLAG (rabbit, CST, 2368S, 1:2000 for WB, 1:500 for IF), anti-pan 14-3-3 [rabbit, CST, 8312S, 1:1000]<br><br>The following secondary antibodies were used: anti-IgG Veriblot (Abcam, ab131366, 1:500), AlexaFluor 488 anti-rabbit IgG (donkey, Life Technologies, A21206, 1:1000
Validation	All antibody validation statements can be found on the manufacturer's websites by searching the specific catalog number specified in the antibodies used section. FLAG and HA antibodies were further validated using empty vector controls.

Eukaryotic cell lines

Policy information about cell lines

Cell line source(s)	Expi293F (Thermo Fisher Scientific), HEK293 (American Type Culture Collection [ATCC]), COS-7 (ATCC)
Authentication	Cells were not authenticated.
Mycoplasma contamination	All adherent cell lines were tested quarterly for mycoplasma contamination using the MycoAlert mycoplasma detection kit (Lonza) and tested negative.
Commonly misidentified lines (See ICLAC register)	No commonly misidentified lines were used in this study.
